# Development of critical K dilution curves for diagnosing sweetpotato K status

**DOI:** 10.3389/fpls.2023.1124328

**Published:** 2023-08-04

**Authors:** Weichen He, Jing Li, Yanjun Lu, Shaojie Chen, Lijuan Deng, Ximing Xu, Yueming Zhu, Minghuan Jin, Yuheng Liu, Guoquan Lu, Zunfu Lv

**Affiliations:** ^1^ The Key Laboratory for Quality Improvement of Agricultural Products of Zhejiang Province, College of Advanced Agricultural Sciences, Zhejiang A&F University, Hangzhou, China; ^2^ Zhejiang Agricultural Technology Promotion Center, Hangzhou, Zhejiang, China; ^3^ Agricultural and Forestry Technology Promotion Center in Lin’an District, Hangzhou, Zhejiang, China; ^4^ Ningbo Agricultural Technology Promotion Station, Ningbo, Zhejiang, China

**Keywords:** sweetpotato, critical K concentration, K nutrition index, source sink balance, K status

## Abstract

Scientific and reasonable application of potassium fertilizer is an important agronomic measure to achieve high yield and high quality of sweetpotato, and it is of great significance to determine the appropriate amount of potassium fertilizer in the field. For this we constructing a model of the critical K dilution curve (CKDC) of sweetpotato under different N levels to determine crop nutritional statuses. In this study, a 3-year field experiment was conducted in Zhejiang Province in China, using two nitrogen levels (N0: 0 kg ha^−1^ and N1: 120 kg ha^−1^) and five K fertilization rates (K0: 0, K1: 75, K2: 150, K3: 225, K4: 300 kg ha^−1^) for two sweetpotato cultivars of ‘Shang 19’ and ‘Yan 25’. Plant dry matter first increased and then decreased and the K concentration increased continuously with an increase in K application rate. The required amount of K fertilizer to achieve maximum sweetpotato yield under high N conditions was greater than that under low nitrogen conditions. A new CKDC based on dry matter and K concentration was created to assess K nutrition in sweetpotato. At two N levels, CKDC was expressed by the negative power function equation, aboveground: K_c(N0)_=*5.30W^-0.463^*, R^2^ = 0.79, and K_c(N1)_=*4.23W^-0.298^*, R^2^ = 0.78, under-ground: K_c(N0)_=*1.38W^-0.125^*, R^2^ = 0.81, and K_c(N1)_=*1.32W^-0.132^*, R^2^ = 0.72;whole-plant: K_c(N0)_=*4.31W^-0.421^*, R^2^ = 0.80; Kc_(N1)_=*3.89W^-0.415^*, R^2^ = 0.79. There is no significantly different for CKDC of whole-plant and underground between N0 and N1 levels, while there is significantly different for CKDC of aboveground between N0 and N1 levels. N fertilizer can strengthen the dilution effect of K concentration, and its effect on the aboveground is greater than that on the underground and whole-plant. Then, potassium nutrition indexes were constructed to identify K nutrition status and could be used as a reliable indicator for K nutrition diagnosis of sweetpotato. The results provide a theoretical basis to improve K fertilization management and sustainability of sweetpotato.

## 1 Introduction

Sweetpotato (*Ipomoea batatas* [L.] Lam.), is one of the main food crops in China; it is extensively planted in tropical and subtropical regions worldwide. Potassium (K), is one of the three major plant nutrients and the most abundant metal cation in plants, comprises 10% of plant dry matter (Zhang et al., 2010). K has important roles in plant growth and yield. K deficiency leads to yellow edges of crop leaves, followed by necrotic death that affects crop yield (Zörb and Senbayram, 2014; Johnson et al., 2022). Previous studies have shown that initial soil K content insufficient limit crop growth and nutrient accumulation (Ali et al., 2019). Appropriate K application can promote the accumulation of dry matter of sweetpotato, increase the rate of starch and sugar accumulation, promote the expansion of storage roots, and prolong the storage period (Chai et al., 2018). However, excessive K application inhibits tuber dry matter accumulation and yield (Chen et al., 2015), leading to decreased tuber quality and yield (Zhao et al., 2017).

In 1984, Lemaire et al. (1984) proposed the concept of critical N concentration in plant dry matter. This concept corresponds to the minimum concentration of nutrient needed by the crop to reach maximum growth rates at a given rate of biomass accumulation (Greenwood et al., 1990). In the field of agricultural research, critical nutrient concentration is an important method for crop nutrition diagnosis. The method for construction of the critical K concentration curve (CKDC) is identical to the method for construction of the critical N concentration curve (Gómez et al., 2018). The CKDC are established based on the concentrations of K (%) of organs and the related dry matter (W). K_c_ could be presented by a negative exponential model: K_c_=*aW^−b^*. The critical K concentration models were generally constructed on root crops in previous researches. Unlike other crops, root crops have a higher demand for potassium, and their harvesting organs are underground parts. A large amount of carbohydrates was transported from stems and leaves to underground storage organs, while potassium nutrition was transported from the underground root to the aboveground parts. Therefore, it is necessary to compare the accuracy and stability of critical K concentration models constructed in different organs.

N promotes the production of photosynthate, whereas K promotes nutrient transport. This interaction between N and K is important for the growth of sweetpotato (Ding et al., 2008). Previous studies have shown that N and K imbalances affect plant K nutrition assessment and fertilization application, and the K concentration of plant affected by different N levels (Li et al., 2022). It is unclear whether N will affect the critical K concentration curve. We needs to identify whether there were differences in the critical K concentration curves under different N modes. In addition, appropriate diagnostic methods in plants can improve nutrient efficiency and reduce nutrient losses in the environment (Bélanger et al., 2001; Abdallah et al., 2016). Gómez et al. (2018) constructed a potassium nutrient index (KNI) for potatoes based on the critical K concentration dilution curve. KNI also was used for K diagnosis for cassava (Geraldine et al., 2020). At present, there were few studies on KNI based on critical K concentration dilution curve. The KNI was effective tool to diagnose the K nutrient status of sweetpotato, and guide the application of fertilizers.

Therefore, the objectives of this study were: (1) To explore the optimal organ for constructing CKDC among aboveground, underground and whole plants of sweetpotato; (2) analyse the changes in CKDC of sweetpotato under different N levels, and clarify the mechanism of nitrogen-potassium interaction;and (3) construct a K nutrition index (KNI) to evaluate K nutritional status. The results will provide theoretical basis for K fertiliser during the management of sweetpotato.

## 2 Materials and methods

### 2.1 Site, experiments and experimental design

This experiment was conducted over three years (2019-2021) at the Zhejiang Agriculture and Forestry University Research Farm in Lin’an District, Hangzhou City, Zhejiang Province, China (30° 15’ 43’, 119° 43’ 25’) on a sandy loam soil. This region has a subtropical monsoon climate, with an altitude of 20–30 m above sea level and an average annual precipitation of 1435 mm. The monsoon season spans June to September. Climate change in 2019 was close to the average of the past decade. In 2020, precipitation was lower than previous year in July and August due to the influence of monsoon weather. In 2021, precipitation was higher than previous year in July and August due to the impact of typhoons. This experiment was conducted at different locations in Lin’an to avoid any potential residual effects of potassium fertilization (**Table 1**). The previous crop was planted with winter wheat.

**Table 1:** Sites, cultivars, fertilizer treatment and soil data for the three field experiments.

Experiment	Site	Cultivar	N levels	K rates	Sampling date	Soil total N (g kg^-1^)	Soil available P (mg kg^-1^	Soil available K (mg kg^-1^)
No			(kg ha^-1^)	(kg ha^-1^)	(Days after transplanting)			
Experiment	Lin’an (30°15′N,	Xinxiang	N0(0)	K0(0) K1(75)	38,54,71,87,	1	24.2	116
1 2019	119°43′E)			K2(150)	104			
				K3(225)				
				K4(300)				
Experiment	Lin’an	Yan 25 (Y25)	N0(0)	K0(0) K1(75)	30,47,61,76,	0.73	22.6	131
2 2020	(30°15′N,	Shang 19 (S19)	N1(120)	K2(150)	92,112			
	119°43′E)			K3(225)				
				K4(300)				
Experiment	Lin’an (30°15′N,	Yan 25 (Y25)	N0(0)	K0(0) K1(75)	30,50,68,88,	0.75	37.6	121
3 2021	119°43′E)	Shang 19 (S19)	N1(120)	K2(150)	105,122			
				K3(225)				
				K4(300)				

N, nitrogen; P, phosphorus; K, potassium.

### 2.2 Field management

The experiment 2 and 3 were conducted from 2020-2021 at the Zhejiang Agriculture and Forestry University Research Farm in Lin’an, Zhejiang Province in China, using two nitrogen levels (N0: 0 kg ha^−1^ and N1: 120 kg ha^−1^) and five K fertilization rates (K0: 0, K1: 75, K2: 150, K3: 225, K4: 300 kg ha^−1^) for two cultivars of ‘Shang 19’ and ‘Yan 25’. The experiment 1 was conducted at same site in 2019 using one nitrogen levels (N0: 0 kg ha^−1^) and five K fertilization rates (K0: 0, K1: 75, K2: 150, K3: 225, K4: 300 kg ha^−1^) for one cultivar of ‘Xinxing’ (**Table 1**). **Table 1** lists the soil total N, available potassium (K), and available phosphorus (P) for the three experiments. Urea and potassium sulfate are all applied as basal fertilizers to all plots. P_2_O_5_ (60 kg ha^-1^) fertilizers were also basally incorporated in all plots before planting as monocalcium phosphate (Ca(H_2_PO_4_)_2_) fertilizer. Three field experiments each processing were conducted using plot sizes of 6×4 m; plots were arranged in a randomized block design with three replicates. The planting density was 5.0 plants m^−2^, row spacing was 25 cm. All the treatment are covered with mulch film, and the water source is natural irrigation. During the trial, all field management, including herbicide application and pest control, was carried out using local cultivation and management methods. The sampling dates are shown in **Table 1**.

### 2.3 Plant sampling and measurements

In 2019, transplant was carried out on June 9 and harvest was performed on September 8. In 2020, transplant was carried out on June 16 and harvest was performed on October 8. In 2021, transplant was carried out on May 14 and harvest was performed on September 22. Plant samples were collected from each treatment on 6 sampling dates during the whole growth period of sweetpotato (**Table 1**). At each sampling date in each plot, five complete plants were harvested from each treatment. Each plant sample was complete divided into three sections: stems, leaves, and underground parts (including tubers roots and fibrous roots). Three bags of each clean fresh sample were randomly selected, each bag of 200g. (the underground part was washed and air dried). Samples were inactivated at 105 °C of dry oven for 30 min, and then dried for 3 days at 80°C of dry oven, measurement of each sample dry matter and calculated the dry rate. The dried tissues matter were ground in a hammer whirlwind grinding mill to pass a 100-mesh sieve, then powder (0.20 ± 0.01 g) was weighed, digested with concentrated H_2_SO_4_ and 30% H_2_O_2_ (Jones and Case, 1990), and analyzed for K nutrient content by using a flame spectrophotometer (FP6431) (Cate and Nelson, 1971).

### 2.4 Critical K dilution curve and KNI

**Figure 1:**
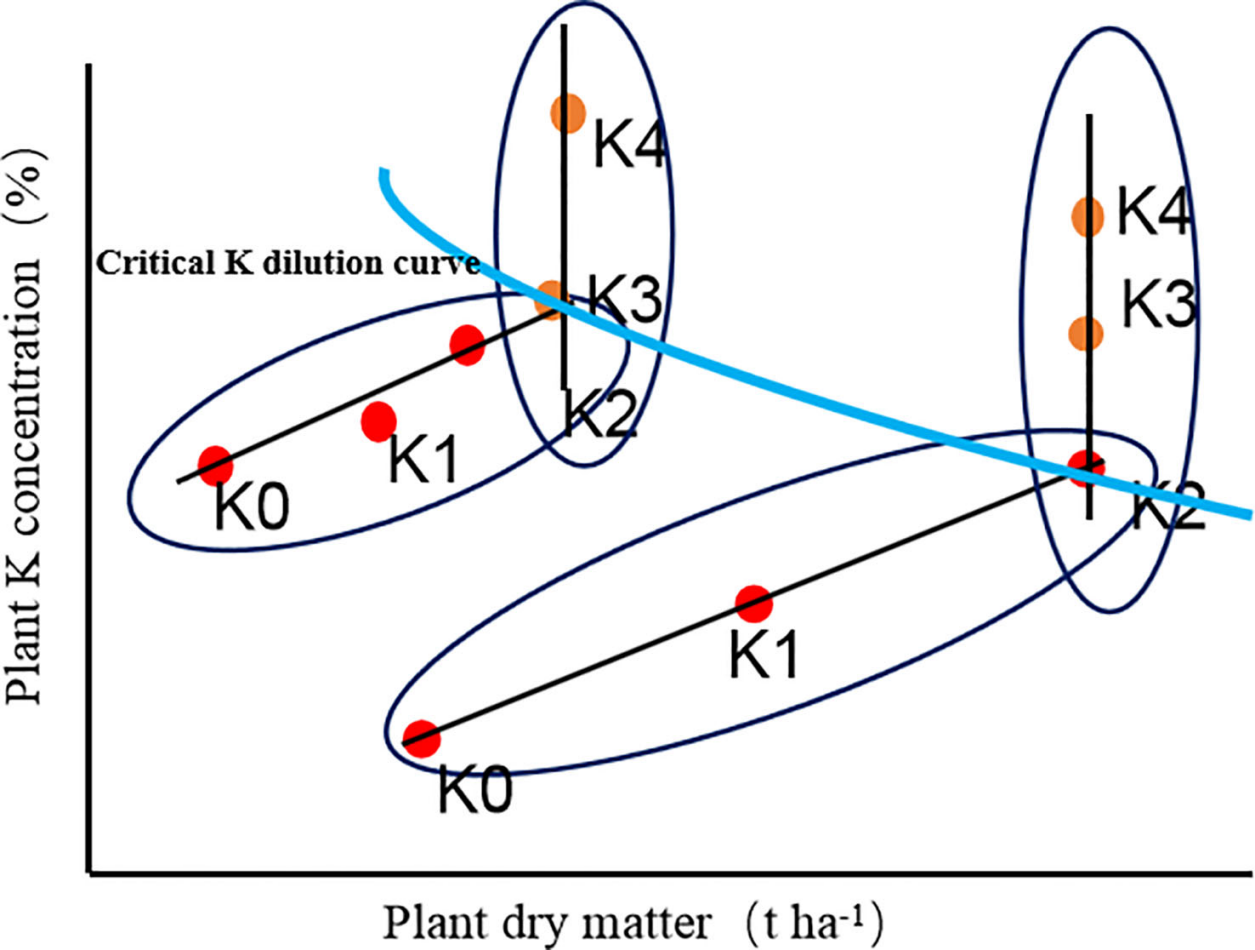
Plant K concentration versus dry matter. Dots represented the measured data in the different K treatments. Red dots: increasing K supply led to significant increases of plant weight and K concentration; Yellow dots: increasing K supply led to a significant response of plant K concentration but not plant weight. Blue line: Critical K dilution curve.


A critical K dilution curve for the whole-plant of sweetpotato was constructed in accordance with the method of the critical N dilution curve (Justes, 1994) was as follows (**Figure 1**):

1) The plant dry matter of each sample under the different K rates were analysed based on the least significant difference (LSD) at the 90% significance level. The plant dry matter and K concentrations were used to determine whether plant growth was restricted by K nutrition. The data points of each sampling date were divided into K-limited treatment group and non-K-limited treatment group. The increase of potassium supply in K-limited treatment resulted in a significant increase in potassium concentration and dry weight, while the increase of potassium supply in non-K-limited treatment resulted in a significant response to potassium concentration, but no significant response to plant weight;
2) An oblique line was used to fit the relationship between plant dry matter and K concentration for K-limited treatment group at each sampling date;
3) A vertical line was used to represent the average of plant dry matter for non-K-limited treatment group at each sampling date;
4) The intersection coordinates of the oblique line and the vertical line was critical K concentration point at each sampling date;
5) A regression line was used to fit data from the critical potassium concentration points. The following power exponential equation describes the CKDC based on the maximum dry matter:

$$K_{c}=aW{\;}^{-b}$$

In the formula: K_c_ (%) was plant critical K concentration, *W* is the plant dry matter (t ha^-1^), where coefficient a represents the critical K concentration when W is 1 t ha^-1^, and parameter b represents a “dilution coefficient”, which describes reduction in nutrient concentration associated with increment in whole-plant dry matter. The combined CKDC model was re-fitted one curve based on the critical K concentration point values constructed in the same organ sites for different cultivated species.

The accuracy of the model was evaluated by root mean square error (RMSE) and normalized root mean square error (n-RMSE), a common method used to identify the fitness of observed and estimated values. we used the RMSE model and independent validation data for validation. A smaller RMSE value indicated better consistency between the simulated value and the measured value, which implied better prediction accuracy. (Jamieson et al., 1991). If n-RMSE< 10%, model stability was considered excellent; if 10%< n-RMSE< 20%, model stability was considered good; if 20%< n-RMSE< 30%, model stability was considered poor.


$$\text{RMSE}=\frac{\sqrt{\sum_{\text{i}=1}^{\text{n}}\;{\left({\text{P}}_{\text{i}}-{\text{Q}}_{\text{i}}\right)}^{\;2}}}{\text{n}}$$

$$\text{n}-\text{RMSE}=\frac{\text{RMSE}}{\text{S}}\;\times 100\%$$

Where n is the number of samples, Pi is an estimated value from the model, Qi is an observed value, and S is the averaged observed value.

The KNI is an indicator used to determine crop K status (Lemaire et al., 2007; Lv and Lu, 2021). The KNI at each sampling date was determined according to the actual K concentration (K_a_) and the critical K concentration (K_c_), as follows:


$$\text{KNI}={\text{K}}_{\text{a}}/{\text{K}}_{\text{c}}$$

Thus, for KNI=1, K nutrition is optimal to obtain maximum biomass; for KNI< 1, K nutrition is limited obtain maximum biomass; and for KNI > 1, K nutrition is excessive obtain maximum biomass.

### 2.5 Data analysis

Excel, SPSS, and Curve Expert software were used for statistical and curve fitting analyses to build a quantitative model. Analysis of variance (ANOVA) was used to compare experimental groups using SPSS ver. 19. The differences dry matter values were in aboveground, under-ground and whole-plant between different K treatments were assessed using the least significant difference test (LSD, *P*< 0.05). The data from Experiments 1-3 were divided into K-limiting and non-K-limiting treatments using the LSD test (*P*< 0.1). The data of Experiments 2 and 3 conducted at each sampling date during the 2020 and 2021growing seasons were combined to construct the critical K dilution curve. The data of Experiment 1 conducted during the 2019 growing season were used to validate the CKDC. The differences in the critical K curves for the whole plant, aboveground and under-ground parts between the two N levels was compared using ANOVA at the 5% probability level.

## 3 Results

### 3.1 Changes in aboveground dry matter and K concentration in sweetpotato

**Figure 2:**
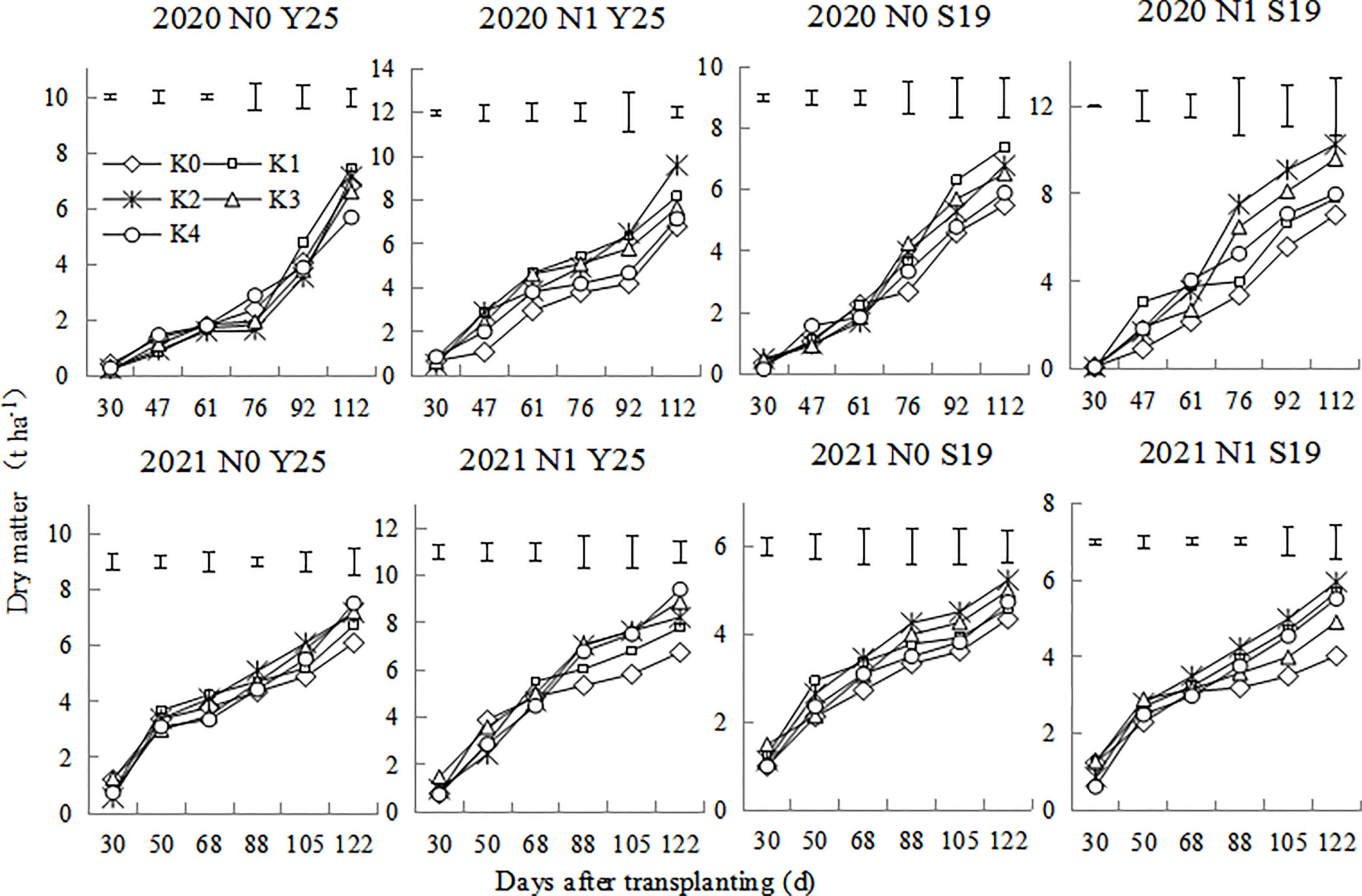
Changes in aboveground dry matter of ‘Yan 25’ and ‘Shang 19’ after planting. Vertical lines in the figure are the minimum non-significant differences per treatment, according to the LSD test, *P*< 0.05.

The aboveground dry matter of sweetpotato plants includes leaves and stems. As shown in the **Figure 2**, the aboveground dry matter of sweetpotato plants gradually increased after transplanting. Dry matter increased rapidly at the early stage, whereas it increased slowly at root expansion stage. There were significant differences for aboveground dry matter for the two cultivars under different K treatments. the aboveground dry matter in 2020 was significantly higher than that in 2021. In 2020, the dry matter was the highest at K1 treatment under the N0 mode, while the dry matter was the highest at the K2 treatment under N1 mode. In 2021, the dry matter was relatively higher at K2 treatment under the N0 mode, while the dry matter was relatively higher at the K2 and K3 treatment under N1 mode.

The plant K concentration increased with increasing K application during each growth period, and there were significant differences among the five K treatments (**Table 2**). These findings indicate that increased application of K fertilizer within a specific range can improve the K concentration in shoots. However, the K concentration in the aboveground parts followed a downward trend throughout the growth period, decreasing rapidly from 30 to 60 days after planting. From 68 to 88 day after planting in 2021, dry matter accumulation significantly increased, whereas the K concentration in the aboveground parts follow a slowly decreasing tread.

**Table 2:** Changes in aboveground K concentration for two sweetpotato cultivars at two N modes and five K rates in two growing seasons.

Year	Cultivars	DAP	Aboveground K concentration (%)									
	N levels		N0					N1				
	K rates		K0	K1	K2	K3	K4	K0	K1	K2	K3	K4
2020	Yan	30	3.40d	3.48c	3.67b	3.70b	3.96a	3.20d	3.40c	3.60b	3.85a	3.89a
	25	47	3.20c	3.15c	3.30b	3.35b	3.60a	2.80d	3.25b	3.03c	3.30b	3.56a
		61	2.68c	3.03b	3.10b	3.20b	3.48a	2.40bc	2.35c	2.54b	2.53b	2.60a
		76	2.49c	2.50c	2.50c	2.79b	3.03a	2.18c	2.18c	2.40b	2.47a	2.19c
		92	2.33d	2.49b	2.45b	2.78a	2.36c	2.10d	2.15c	2.30b	2.39a	2.30b
		112	2.00d	2.48b	2.3	2.68a	2.39c	1.90d	1.96c	2.00b	2.18a	2.20a
	Shang	30	4.00d	4.13b	4.20c	4.40b	4.50a	3.45d	3.63c	3.74b	3.74b	3.98a
	19	47	3.93b	3.76c	3.75c	3.70d	3.82a	3.27d	3.33c	3.52b	3.57b	3.78a
		61	3.21d	3.33b	3.40c	3.50b	3.61a	3.00d	3.00d	3.29c	3.54b	3.65a
		76	3.13b	2.69d	2.70d	3.50a	3.17b	2.77d	2.90b	3.29a	2.86c	2.99b
		92	2.94b	2.45d	2.53c	2.65c	3.02a	2.80c	2.83c	3.05b	2.58d	3.10a
		112	2.50c	2.23d	2.79c	2.95b	3.00a	2.45d	2.56d	2.78c	2.89b	3.13a
2021	Yan	30	2.93c	3.04c	3.66b	4.54a	4.30b	3.34b	3.35b	3.14c	3.51b	4.44a
	25	50	2.80d	2.90c	3.41a	3.00b	2.99b	2.32c	2.44bc	2.41b	2.97b	3.57a
		68	2.68c	2.64d	2.91b	3.02a	3.05a	1.98c	2.58b	2.76b	3.04b	3.25a
		88	2.55b	2.15c	2.90a	2.97a	3.03a	1.70c	2.18a	1.98b	2.05b	2.32a
		105	2.68a	2.45b	2.57a	2.52b	2.03c	1.52c	1.99b	1.75b	2.05b	2.57a
		122	1.82d	2.30b	2.62a	2.71a	1.88c	1.68d	2.56b	2.19c	2.04c	3.06a
	Shang	30	3.24c	3.52b	4.01a	4.03a	3.83a	2.66c	2.63c	3.15b	3.22b	3.68a
	19	50	2.93c	3.29c	3.16b	3.54a	3.34b	2.43c	2.36c	2.47b	2.58b	2.82a
		68	3.00a	2.73b	2.76b	2.83b	2.60b	2.07c	2.06c	2.25b	2.63a	2.55b
		88	2.02c	2.84a	2.10c	2.25b	2.44b	1.93b	1.94b	1.93b	2.55a	1.97b
		105	1.78c	2.53a	2.00b	2.30b	2.13b	1.60d	1.64c	1.87b	2.10a	1.68c
		122	1.93c	2.94a	2.32b	1.99c	2.54b	1.85b	1.62c	2.05a	2.12a	1.91b

Different small letters in the same column indicate significant differences between K treatments at the same N application rate (P<0.05).

### 3.2 Changes in underground dry matter and K concentration in sweetpotato

**Figure 3:**
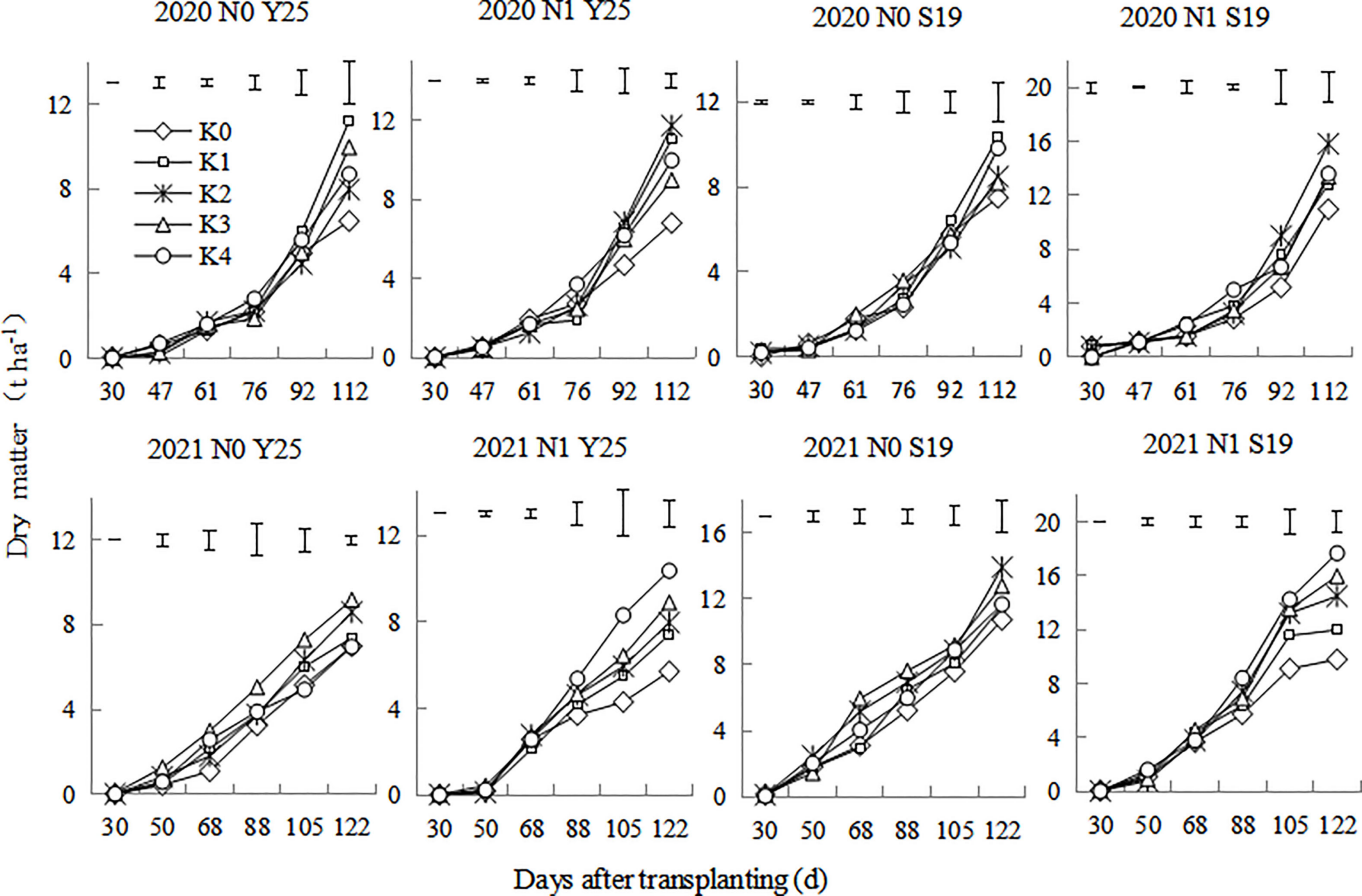
Changes in the underground dry matter of ‘Yan 25’ and ‘Shang 19’ after planting. Vertical lines in the figure are the minimum non-significant differences per treatment, according to the LSD test, P< 0.05.

Under different K rates, the underground dry matter of sweetpotato increased under the whole growth period. As shown in the **Figure 3**, the underground dry matter increased slowly from 30 to 76 days after planting, dry matter increased rapidly from 76 to 112 days after planting. In 2020, ‘Shang 19’ and ‘Yan 25’ had the highest underground dry matter at K1 treatment under the N0 mode, while ‘Shang 19’ and ‘Yan 25’ had the highest underground dry matter at K2 treatment under the N1 mode. In 2021, ‘Shang 19’ and ‘Yan 25’ had relatively higher underground dry matter at K2 and K3 treatment under the N0 mode, while ‘Shang 19’ and ‘Yan 25’ had the highest underground dry matter at K4 treatment under the N1 mode. Moderate application of K promoted underground growth, but excessive application of K inhibited underground growth.

The K concentration in underground parts increased with increase of K application, and there were significant differences among the five K treatments (**Table 3**). The K concentration in the underground parts followed a downward trend throughout the growth period. The K concentration in the underground parts decreased rapidly from 30 and 68 days after planting. From 68 days after planting, the K concentration in the underground parts decreased slowly.

**Table 3:** Changes in underground K concentration for two sweetpotato cultivars at two N modes and five K rates in two growing seasons.

Year	Cultivars	DAP	Underground K concentration (%)									
	N levels		N0					N1				
	K rates		K0	K1	K2	K3	K4	K0	K1	K2	K3	K4
2020	Yan	30	1.37c	1.42b	1.43b	1.44b	1.53a	1.27c	1.28c	1.27c	1.3b	1.33a
	25	47	1.11c	1.32b	1.27b	1.10c	1.30a	1.09c	1.18b	1.16b	1.12b	1.19a
		61	1.14b	1.11b	0.94c	1.13b	1.26a	0.90d	1.08a	1.05b	0.95c	1.04b
		76	0.81c	0.93a	0.94a	0.83b	0.93a	0.73c	0.86b	0.85b	0.84b	0.97a
		92	0.84b	0.92a	0.9a	0.78c	0.84b	0.65d	0.78c	0.75c	0.81b	0.85a
		112	0.83b	1.00a	0.73c	0.67d	0.92a	0.68b	0.57d	0.66c	0.83b	0.84a
	Shang	30	1.45d	1.53c	1.56b	1.66a	1.59a	1.21c	1.24c	1.34b	1.42a	1.45a
	19	47	1.34d	1.33d	1.39c	1.43b	1.52a	1.18d	1.20c	1.30a	1.22b	1.35a
		61	1.28b	1.23c	1.30a	1.30a	1.29a	1.10c	1.15b	1.02d	1.19a	1.23a
		76	1.03b	0.85d	0.94c	1.03b	1.05a	0.68b	0.64b	0.90a	0.98a	1.01a
		92	0.65d	0.93b	0.87c	0.86c	0.94a	0.68c	0.75b	0.94a	0.96a	0.73b
		112	0.87d	0.87d	0.97c	1.02b	1.08a	0.83c	0.96b	0.74d	0.96a	1.12a
2021	Yan	30	1.52c	1.57c	1.52c	2.37a	2.26b	1.52c	1.76b	1.87a	1.78b	1.74b
	25	50	1.40b	1.07c	1.07c	1.43a	1.39b	1.22a	1.11c	1.24a	1.16b	1.24a
		68	0.99b	1.09a	1.01b	1.09a	0.99c	1.16a	0.81b	0.83b	0.85b	0.77c
		88	0.83b	0.79b	0.87b	0.87b	0.90a	0.91a	0.68c	0.61c	0.81b	0.77b
		105	1.12a	1.09a	0.96c	1.09a	1.03b	1.04a	0.86b	0.59c	1.01b	0.65b
		122	0.98b	1.22a	1.25a	1.26a	1.04b	1.03b	0.99b	0.68c	1.31a	1.01b
	Shang	30	1.37c	1.48b	1.57b	1.81a	1.43a	1.17c	1.30b	1.24b	1.21b	1.44a
	19	50	1.05b	1.25b	1.29a	1.39a	1.43a	0.99b	0.99b	1.00a	1.04a	1.00a
		68	1.08c	1.13a	1.09b	1.14a	1.05c	0.79c	0.74c	0.99b	1.16a	0.90b
		88	0.68c	0.99a	0.82b	0.98a	0.94a	0.81b	0.82b	1.05a	1.07a	0.72c
		105	0.68c	1.01a	0.96a	0.70c	0.91b	0.69c	1.04a	0.64d	0.82b	0.85b
		122	0.66c	1.12a	0.85b	0.85b	0.85b	0.72c	0.74c	0.75bc	0.85a	0.61d

Different small letters in the same column indicate significant differences between K treatments at the same N application rate (P<0.05).

### 3.3 Changes in whole-plant dry matter and K concentration in sweetpotato

**Figure 4:**
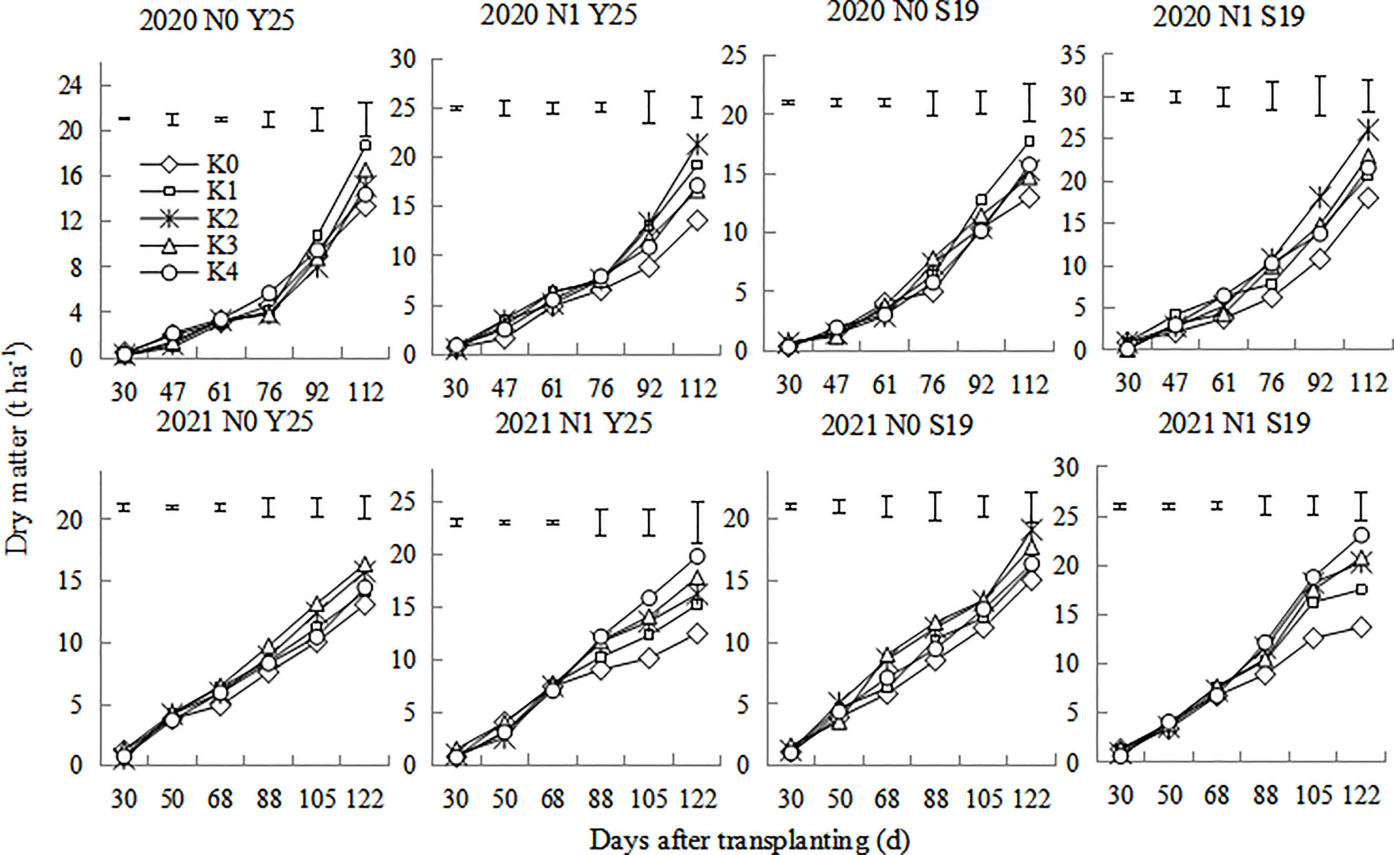
Changes in whole-plant dry matter of the ‘Yan 25’ and ‘Shang 19’ varieties after planting. Vertical lines in the figure are the minimum non-significant differences per treatment, according to the LSD test, *P*< 0.05.

The whole-plant dry matter of sweetpotato gradually increased after planting (**Figure 4**). Dry matter rapidly increased during 70-100 days after planting. There were significant differences for plant dry matter of the two cultivars under different K treatments. The plant dry matter in 2020 were slightly higher than that in 2021. The dry matter under the N1 mode for both cultivars was significantly higher than that under the N0 mode. In 2020, the dry matter was the highest at K1 treatment under the N0 mode, while the dry matter was the highest at the K2 treatment under N1 mode. In 2021, the dry matter was relatively higher at K2 and K3 treatment under the N0 mode, while the dry matter was the highest at the K4 treatment under N1 mode.

As shown in **Table 4**, there were significant differences for K concentration under different K treatments. K concentration consistently decreased during the growth period of sweetpotato. K concentration decreased significantly for K3 and K4 treatment at the early stages. The overall K concentration of ‘Shang19’ is higher than that of ‘Yan 25’. The variation range of K concentration for two cultivars is from 0.76% to 4.38%. K content tended to decrease after planting, whereas plant dry matter tended to increase with decreasing K concentration.

**Table 4:** Changes in whole-plant K concentration for two sweetpotato cultivars at two N modes and five K rates in two growing seasons.

Year	Cultivars	DAP	Whole-plant K concentration (%)									
	N levels		N0					N1				
	K rates		K0	K1	K2	K3	K4	K0	K1	K2	K3	K4
2020	Yan	30	3.10d	3.20c	3.48b	3.45b	3.70a	3.11d	3.24c	3.45b	3.71a	3.72a
	25	47	2.54c	2.92a	2.85b	2.89b	2.85b	2.23c	2.86b	2.74b	2.95b	3.05a
		61	2.02d	2.19c	1.98d	2.27b	2.43a	1.80c	2.01a	2.17a	2.10a	1.98b
		76	1.86b	1.64c	1.61c	1.83b	2.00a	1.57d	1.84b	1.86b	1.94a	1.62c
		92	1.51b	1.61a	1.59b	1.65a	1.46b	1.38d	1.45c	1.80a	1.40c	1.50b
		112	1.43c	1.59a	1.47b	1.47b	1.50a	1.33c	1.16d	1.26	1.45b	1.70a
	Shang	30	3.33c	3.20d	3.48b	3.35c	3.60a	2.50c	2.89b	3.30a	3.39a	3.69a
	19	47	2.98c	3.10b	2.95c	3.12b	3.34a	2.11d	2.76b	2.67b	2.65b	2.86a
		61	2.36c	2.56b	2.50b	2.36c	2.65a	2.20d	2.25c	2.59b	2.68b	2.76a
		76	2.15c	1.90d	1.89d	2.37a	2.27b	1.81d	1.80d	2.57a	2.20b	2.03c
		92	1.67c	1.68c	1.71b	1.75b	1.92a	1.78b	1.72b	2.00a	1.85c	1.95b
		112	1.56b	1.44c	1.78a	1.88a	1.80a	1.47d	1.56c	1.54c	1.70b	1.87a
2021	Yan	30	2.82d	2.96c	3.58b	4.38a	4.16a	3.14c	3.24b	3.04c	3.41b	4.30a
	25	50	2.24c	2.64a	2.60a	2.45b	2.64a	2.23b	2.31b	2.29b	2.71b	3.32a
		68	2.13b	1.96c	2.25a	1.91c	1.97c	1.58c	2.02b	1.91b	2.00b	2.22a
		88	1.67c	1.70c	1.76b	1.80b	2.00a	1.35b	1.43b	1.33c	1.33c	1.65a
		105	1.55b	1.40c	1.47c	1.44c	1.70a	1.32b	1.27b	1.00c	1.28b	1.39a
		122	1.02b	1.23c	1.25c	1.27c	1.70a	1.05c	1.44b	1.28b	1.21b	1.62a
	Shang	30	3.03b	3.39b	3.70a	3.72a	3.68a	2.49c	2.52c	2.98b	2.99b	3.93a
	19	50	1.86b	2.43a	1.79b	2.43a	1.93b	2.06d	2.14c	2.58a	2.78a	2.42b
		68	1.70b	1.69b	1.65b	1.59c	1.86a	1.35d	1.49c	1.79b	1.92a	1.78b
		88	0.81d	1.29b	1.09b	1.19c	1.31a	1.30b	1.23c	1.32b	1.64a	1.36b
		105	0.78c	1.21a	1.04b	1.03b	0.92b	0.85c	1.07a	0.94b	1.01b	0.95b
		122	0.76c	1.16a	0.89bc	0.88b	0.91b	0.89b	0.79b	1.05a	0.87b	0.76c

Different small letters in the same column indicate significant differences between K treatments at the same N application rate (P<0.05).

### 3.4 Construction of critical K dilution curve model

#### 3.4.1 Critical K dilution curve model based on aboveground dry matter of sweetpotato

**Figure 5:**
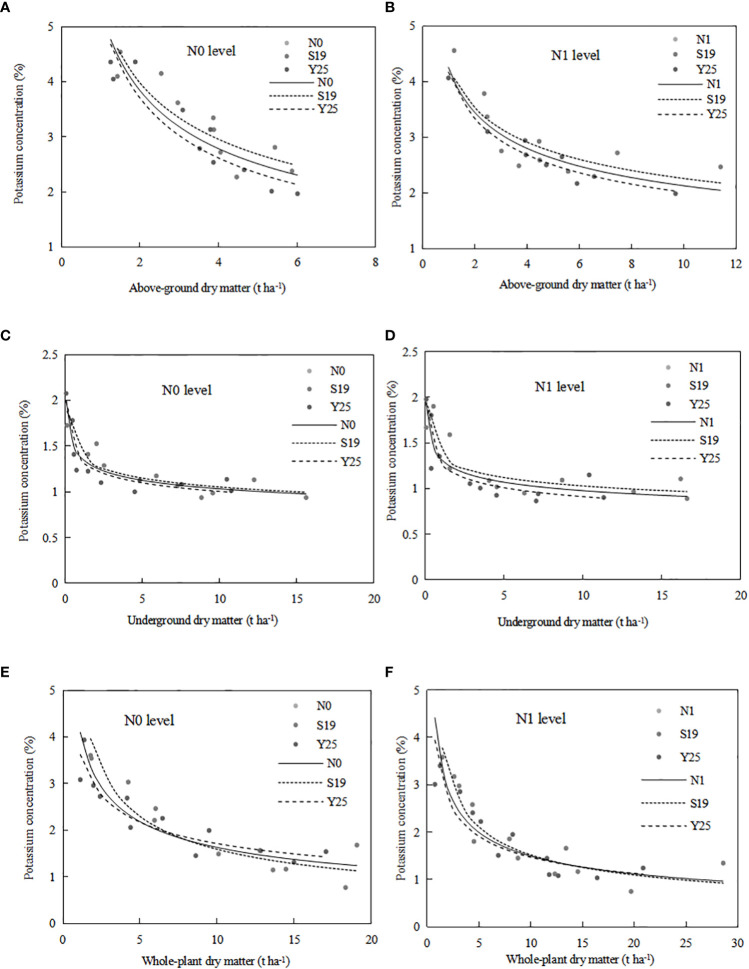
The CKDC for sweetpotato aboveground parts **(A, B)**, underground sweetpotato parts **(C, D)** and whole-plant **(E, F)**. The dashed lines represent the curves for each cultivar, while the solid lines represent the combination curves of the two cultivars.

The critical K dilution curve was constructed based on the aboveground dry matter and K concentration. The aboveground CKDC for ‘Shang 19’ and ‘Yan 25’ were (**Figures 5A, B**):

S19: K_c(N0)_=*5.37W^-0.429^*, R^2^ = 0.78; K_c(N1)_=*4.27W^-0.276^*, R^2^ = 0.69;
Y25: K_c(N0)_=*5.25W^-0.501^*, R^2^ = 0.89; K_c(N1)_ *=4.15W^-0.313^*, R^2^ = 0.94.

Parameter a and b of CKDC under N0 mode is greater than that under N1 mode. Both cultivars had higher maximum aboveground dry matter under the N1 mode than under the N0 mode. The critical K curves of the two sweetpotato cultivars showed a similar trend under the influence of N. The CKDC under N1 mode was lower than that under N0 mode. There is no significant differences for CKDC between ‘S19’ and ‘Y25’, so the critical K curves of two cultivars can be fitted into one curve: K_c(N0)_=*5.30W^-0.463^*, R^2^ = 0.79; K_c(N1)_=*4.23W^-0.298^*, R^2^ = 0.78.

#### 3.4.2 Critical K dilution curve model based on underground dry matter of sweetpotato

Critical K dilution curves for both sweetpotato cultivars were constructed based on underground dry matter and K concentration. The underground CKDC for ‘Shang 19’ and ‘Yan 25’ were (**Figures 5C, D**):

S19: K_c(N0)_=*1.41W^-0.127^*, R^2^ = 0.81; K_c(N1)_=*1.37W^-0.124^*, R^2^ = 0.70;
Y25: K_c(N0)_=*1.35W^-0.129^*, R^2^ = 0.83; K_c(N1)_=*1.27W^-0.147^*, R^2^ = 0.79.

Parameter a of CKDC under N0 mode is greater than that under N1 mode. Both cultivars had higher maximum root dry matter under the N1 mode than under the N0 mode. The CKDC of ‘Shang 19’ under different N levels was slightly higher than the CKDC of ‘Yan 25’. The curve changes were consistent under the N0 and N1 levels in both years, and the CKDC under N1 mode was lower than that under N0 mode. There is no significant differences for CKDC between ‘Shang 19’ and ‘Yan 25’, so the critical K curves of two cultivars can be fitted into one curve: K_c(N0)_=*1.38W^-0.125^*, R^2^ = 0.81; K_c(N1)_=*1.32W^-0.132^*, R^2^ = 0.72.

#### 3.4.3 Critical K dilution curve model based on whole-plant dry matter of sweetpotato

Critical K dilution curves for both sweetpotato cultivars were constructed based on whole-plant dry matter and K concentration. The whole-plant CKDC for ‘Shang 19’ and ‘Yan 25’ were (**Figures 5E, F**):

S19: K_c(N0)_=*5.4W^-0.531^*, R^2^ = 0.82; K_c(N1)_=*4.52W^-0.473^*, R^2^ = 0.80;
Y25: K_c(N0)_=*3.77W^-0.342^*, R^2^ = 0.86; K_c(N1)_=*3.51W^-0.378^*, R^2^ = 0.81.

The **Figure 5** shows that the CKDC was similar for both N levels. Parameter a of CKDC under N0 mode is greater than that under N1 mode. Both cultivars had higher maximum root dry matter under the N1 mode than under the N0 mode. The parameter a and b values for ‘Shang 19’ were slightly higher than that for ‘‘Yan 25’. The curve changes were consistent under the N0 and N1 levels in both years, and the CKDC under N1 mode was lower than that under N0 mode. There is no significant differences for CKDC between ‘S19’ and ‘Y25’, so the critical K curves of two cultivars can be fitted into one curve: K_c(N0)_=*4.31W^-0.421^*, R^2^ = 0.80; K_c(N1)_=*3.89W^-0.415^*, R^2^ = 0.79.

### 3.5 Effects of different N levels on CKDC

As shown in **Table 5**, the CKDC for aboveground parts, underground parts and the whole plant under N1 mode were lower than that under N0 mode, and parameters a under N1 mode were also lower than that under N0 mode. The CKDC for aboveground parts under N1 mode was significantly lower than that under N0 mode at early stage of sweetpotato, and parameters a and b for the aboveground parts were significantly different between N0 and N1 levels (*P*<0.05). The CKDC for underground parts and the whole-plant under N1 mode were slightly lower than that under N0 mode, there is no significant differences for parameters a and b for underground parts and the whole plant between N0 and N1 levels (*P*>0.05). This is duo to the impact of N on aboveground dry matter is significantly greater than that on underground dry matter. The parameters a and b for ‘Shang 19’ were higher than that for ‘Yan 25’.

**Table 5:** Critical K dilution curves based on different parts under N0 and N1 modes.

Parameter	Nutrient level	Dilution curve	Range W t ha^-1^	R^2^	*P*
		K_c_=*aW* ^−^ *^b^*			
Aboveground part	N0	5.30W^-0.463^*	1-6.0	0.79	0.04
	N1	4.23W^-0.298^*	1-11.4	0.78	
Underground part	N0	*1.38W^-0.125^ ns*	1-15.6	0.81	0.50
	N1	*1.32W^-0.132^ ns*	1-16.2	0.72	
Whole plant	N0	*4.31W^-0.421^ ns*	1-19.1	0.80	0.34
	N1	*3.89W^-0.415^ ns*	1-27.6	0.79	

*Significant differences (P<0.05) of dilution curves between N levels; ns, not significant.

### 3.6 Validation of the CKDC for sweetpotato

**Figure 6:**
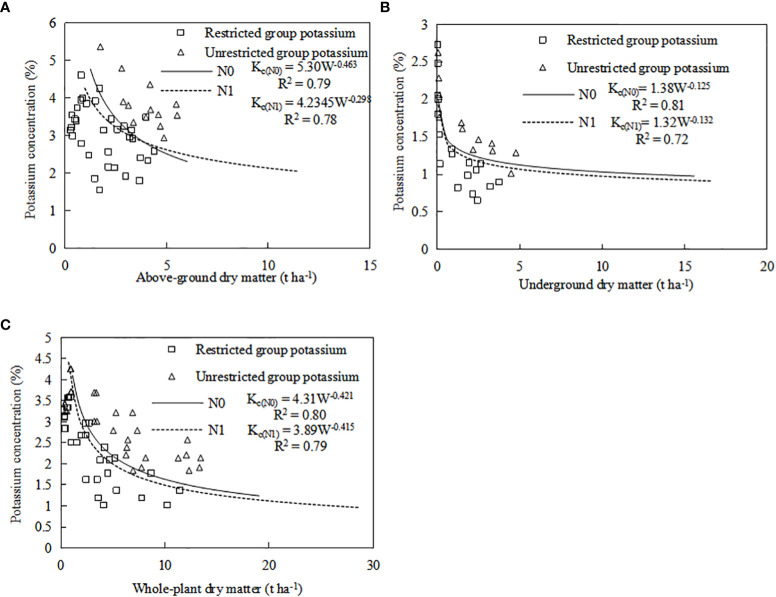
Validation of the CKDC for sweetpotato. **(A)** the combined CKDC for aboveground, **(B)** the combined CKDC for underground, **(C)** the combined CKDC for whole-plant. The △ dot represents excessive K nutrition, £ dot represents K nutrition deficiency, which comes from the independent data of Xin Xiang cultivated in 2019.

A common model was established by combining the CKDC for both sweetpotato cultivars in both years. Independent data of ‘Xin Xiang’ cultivated in 2019 were used for validation. The CKDC could distinguish K-limiting and non-K-limiting groups (**Figure 6**). Almost all the data points limited by K were located below the CKDC, whereas the data points were not limited by K were located above the CKDC (**Figure 6**). Results indicated the curve discriminated well between K-limiting and non-K-limiting treatments. The accuracy of the model was evaluated with RMSE and n-RMSE, by using eqs (**Table 6**). Results showed that the n-RMSE all SD values were less than 10%, indicating ‘good’ agreement between observed and estimated values. As shown in **Table 6**, the CKDC models had high accuracy for both N levels, indicating that they can be used for determination of K nutritional status for sweetpotato. Thus, the W-K_c_ model we established can be used for diagnosis plant nitrogen nutrition.

**Table 6:** Validation of critical K dilution curve for aboveground parts, underground parts and whole-plant.

Dry matter part	N0		N1	
	RMSE	n-RMSE	RMSE	n-RMSE
Aboveground	0.206	1.430	0.197	1.612
Underground	0.143	4.415	0.163	5.214
Whole plant	0.183	5.067	0.135	6.234

n-RMSE< 10%, model stability is excellent.

### 3.7 KNI nutritional status

**Figure 7:**
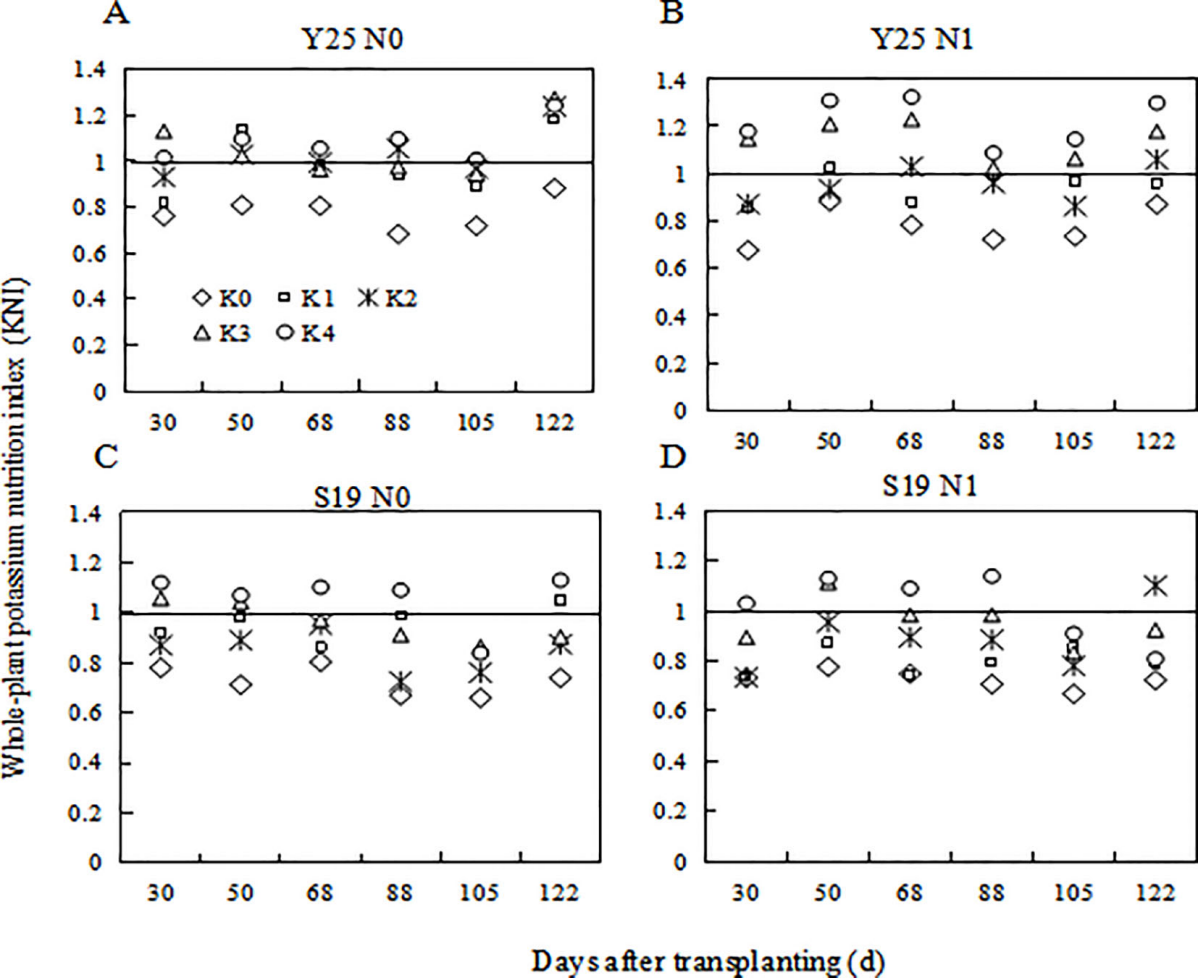
Changes in whole-plant K nutrition index for different K fertilization levels in 2021. **(A)** ‘Yan 25’ N0 level KNI, **(B)** ‘Yan 25’ N1 level KNI, **(C)** ‘Shang 19’ N0 level KNI, and **(D)** ‘Shang 19’ N1 level KNI.

The KNI is used to determine a plant’s real-time K status. The whole-plabt KNI increased with the increase of K application. For the same K treatment, the KNI increased with the increase of N application. When KNI is closer to 1, the plant is in an optimal state of K nutrition to obtain maximum biomass. K1 and K2 were closer to 1 under the N0 level, whereas K2 and K3 were closer to 1 under the N1 level. KNI followed an overall trend of decreasing first and then increasing during plant growth (**Figure 7**).

## 4 Discussion

### 4.1 Comparative analysis of other critical potassium dilution curves

The critical K dilution curves (CKDC) were constructed based on K concentration and dry matter followed a negative exponential model (**Figure 5**). According to the critical dilution curve, the K concentration decreases with the increase of dry matter, which is complying with the “law of dilution” proposed by Justes (1994). Previous reports have shown that the critical K concentration dilution curve of root tuber crops, such as potato (Cogo et al., 2006; Gómez et al., 2018) and cassava (Geraldine et al., 2020). The existing CKDC of cassava in the aboveground part was Kc=*4.2W^-0.690^*, while CKDC of cassava in the whole-plant was K_c_=*4.3W^-0.540^* (Geraldine et al., 2020). The CKDC of three potato cultivars in whole-plant were ‘Asterix’ K_c_=*5.54W^-0.317^*, ‘Suprema’ K_c_=*6.58W^-0.135^*, ‘Capiro’ K_c_=*9.02W^-0.269^*, respectively (Cogo et al., 2006; Gómez et al., 2018).

**Figure 8:**
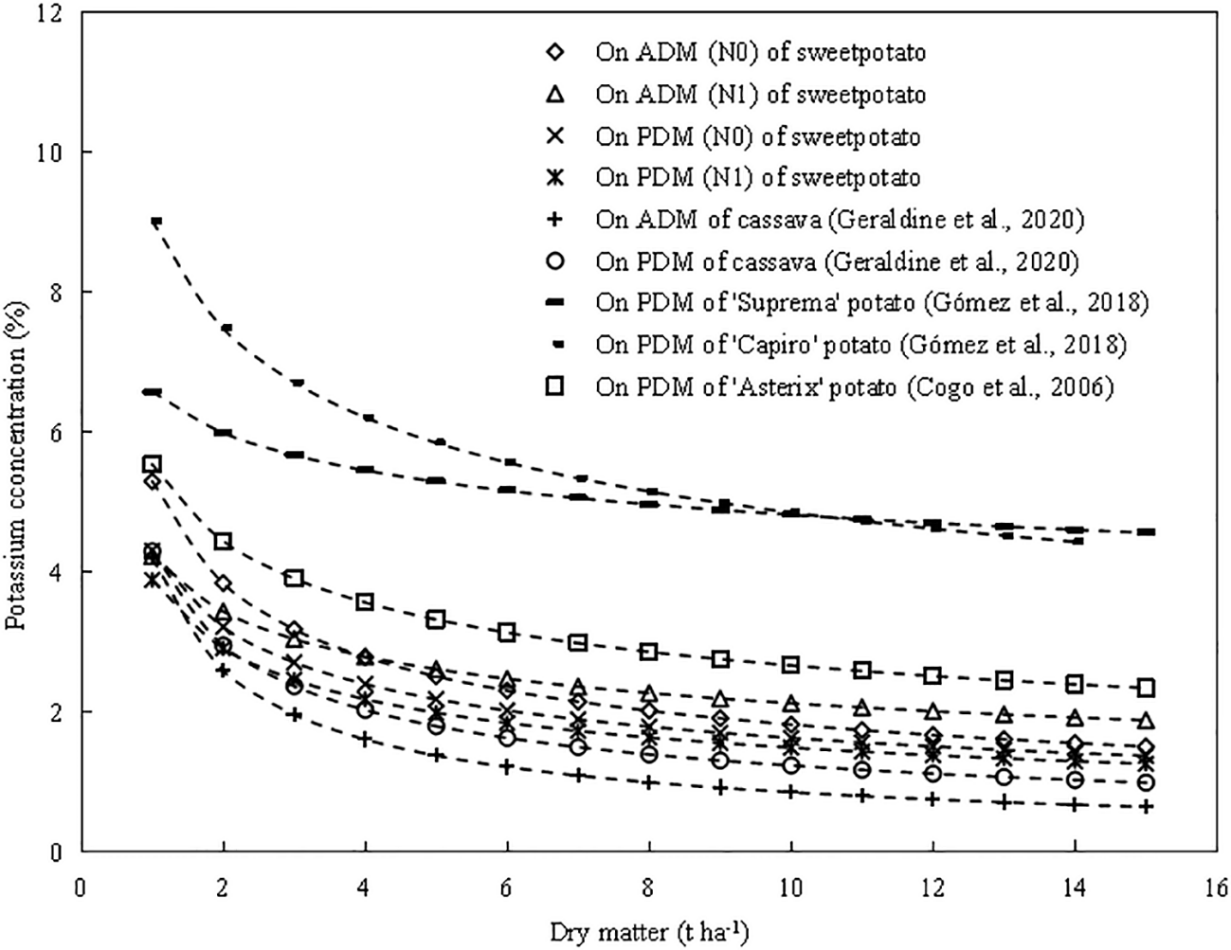
Comparison of different critical potassium (K_c_) curves on sweetpotato, potato and cassava. The K_c_ curve of two N levels sweetpotato based on aboveground and whole-plant dry matter (W) in China.The K_c_ curve of cassava (K=*4.2W^-0.690^*) based on aboveground and (K=*4.3W^-0.540^*) whole-plant dry matter (W) in Brazil (Geraldine et al., 2020). The Kc curve of potato (‘Suprema’ K_c_=*6.58W^-0.135^*, ‘Capiro’ K_c_=*9.02W^-0.269^*) based on whole-plant dry matter (W) in Colombia (Gómez et al., 2018). The Kc curve of potato (‘Asterix’ K_c_=*5.54W^-0.317^*) based on whole-plant dry matter (W) in Brazil potato (Cogo et al., 2006).

The coefficient a represents the value of Kc at the value of dry matter W of 1 t ha^-1^. The coefficient a (4.20) of cassava in whole-plant was lower than that (4.31) of sweetpotato, and the coefficient a of sweetpotato in whole-plant was lower than that (9.02, 6.58, 5.54) of potato (**Figure 8**). For the whole-plant of sweetpotato, potato and cassava species, the dry matter accumulation of cassava was higher than that of sweetpotato, and the dry matter accumulation of sweetpotato was higher than that of potato. The coefficient b (0.69) of cassava in whole-plant was higher than that (0.421) of sweetpotato, and the coefficient b of sweetpotato was higher than that (0.135, 0.269, 0.317) of potato, indicating that the potassium dilution degree of cassava was greater than that of sweetpotato and potato. This difference between species should be related to the intrinsic ratio of carbohydrates and potassium accumulated in the plant based on starch during root expansion growth. The growth cycle of “cassava > sweetpotato > potato”, resulting in higher dry matter accumulation of cassava and sweetpotato. This different growth cycle and dry matter accumulation led to obvious K dilution in cassava. With the progress of crop growth, % Kc gradually decreased at the aboveground and underground levels, and the trend was similar to that of % Kc at the whole plant level (**Figure 8**).

The coefficient a (1.38 and 1.32) of CKDC in underground part was lower than that in the aboveground part (5.3 and 4.23), which was due to the K concentration in aboveground part was higher than that in underground part. The coefficient b (0.125 and 0.132) of CKDC in underground part was lower than that in the aboveground part (0.463 and 0.298), which showed that the decrease of the K concentration in the aboveground was greater than that in the underground. When the plant dry matter is lower than 4 t ha^-1^, there is a significant decrease in shoot potassium concentration (**Figure 8**). This is attributed to the allometric relationship between K concentration and plant W accumulation during the pre-tuberization period of sweetpotato. On the contrary, when the plant dry matter exceeded 4 t ha^-1^, sweetpotato entered the root expansion stage, and the decrease of Kc is more slowly in underground part. This is due to the higher transfer rate of metabolic (leaf) and structural (stem) components of sweetpotato to plant (root) storage components to ensure yield and starch formation (Allen et al., 1976; Sharma and Arora, 1987).

### 4.2 Diagnosis of K nutritional status based on KNI

The critical curves for K_c_ and KNI define a crop’s nutritional status, as reported by Gómez et al. (2017). The KNI can be used to determine the real-time nutritional status of a sweetpotato plant and quantify the fertilizer ratio (He et al., 2017; Soratto et al., 2022), thereby aiding the balanced application of K and N fertilizers. The KNI accurately reflects the K nutrition statuses of potato, cassava, and sweetpotato; it increases as the rate of K application increases. This study constructed CKDC for aboveground, underground and whole-plant, respectively. There was significant difference in CKDC for aboveground parts among different N treatments. Therefore, it is not stable enough for K nutrition diagnosis by using aboveground CKDC to construct KNI. Due to the small biomass in the underground during the early stages of sweetpotato, it may result in significant errors by using underground CKDC for potassium nutrition diagnosis in the early stages. In addition, there is a large amount of dry matter transport between the aboveground and underground parts during the growth period of sweetpotato, which also affects the accuracy of the CKDC of aboveground and underground parts. The CKDC constructed based on the whole-plant of sweetpotato is the most stable, with the highest R^2^, and can be used to diagnose the potassium nutrition status of sweetpotato. Therefore, the KNI constructed based on the whole-plant CKDC can be used to determine K excess or insufficient during plant growth periods.

**Figure 9:**
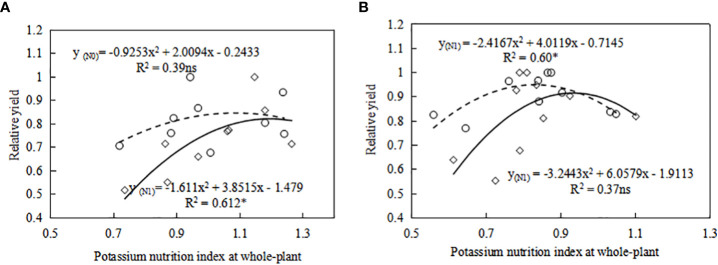
Relationship between relative yield (RY) and whole-plant potassium nutrition index (KNI) for **(A)** Y25 and **(B)** S19 in tuber expansion stage (105-122 DAP). The symbol ○ and solid line represents relationship between RY and KNI at N0 level. The symbol ◇ and dashed line represents relationship between RY and KNI at N1 level. The relative yield is the yield of each treatment divided by the maximum yield.* Significant differences (P<0.05); ns, not significant.

With the increase of K fertilizer application, the KNI value first increases and then decreases. Under the condition of potassium limitation, the yield of sweetpotato depends on the K state of the whole plant, and higher potassium can produce more yield. When KNI was higher than 0.84 for S19 under N0 level, relative yield of sweetpotato had a negative correlation with KNI (**Figure 7**). When KNI was higher than 0.94 for S19 under N1 level, relative yield of sweetpotato had a negative correlation with KNI (**Figure 7**). There is the same trend for Y25. Therefore, with the increase of N fertilizer application, the KNI corresponding to the maximum relative yield also increased, and the threshold for reducing sweetpotato yield caused by K fertilizer application gradually increases. There are some differences in the sensitivity of different cultivars to potassium nutrition (**Figure 9**). Gómez et al. (2017) indicates that extravagant potassium consumption may limit tuber formation and tuber growth, consistent with this study (**Figure 9**). Thus the KNI could be used to assess crop K status and CKDC and to determine the requirement for additional K fertilization using established relationships between the relative yield and KNI.

### 4.3 Interactions between N and K

For aboveground parts of sweetpotato, coefficients *a* and *b* of the CKDC significantly differed under two N levels; there was no significant differences for underground parts. In the initial stage of growth (the first 60-80 days), the K concentration for aboveground parts under N1 treatment was 10.75% lower than that under N0 treatment, while the K concentration in underground parts under N1 treatment was 5.01% lower than that under N0 treatment. These differences were mainly because N fertilizer promotes the growth of leaves and stems at the early stage, and decreased aboveground K concentration. However, the dilution effect of N on the underground parts was weaker than its impact on aboveground parts. In the present study, N showed an increasing dilution effect on the critical K curve and improved KNI. The phenomenon of “law of dilution” through expansive growth was also explained by Lemaire et al. (2007) and Chakwizira et al. (2016).

Sweetpotato has a typical source–sink relationship, and there is a reciprocal relationship between N and K fertilizer. The dry matter of aboveground and whole-plant was more related to fertilization supply as evidenced by the significance of N × K fertilization interaction (**Table 7**). Thus, balanced application of N and K fertilizer is essential to optimize sweetpotato quality and yield. Under the N1 level, the whole-plant CKDC showed greater accumulation of dry matter. High N led to increased whole-plant dry matter and a dilution of the K concentration (Li et al., 2012). Although some studies have shown that high N increases absorption of K, we found that the dilution effect of N on K was greater than the effect of N on K absorption, consistent with the results reported by Wang S et al. (2017).

**Table 7:** Dry matter and K concentrations for Shang 19 and Yan 25 statistical significance of factors and their interactions in 2020-2021.

ANOVA	F Value											
Parameter	Aboveground				Underground				Whole-plant			
	Dry matter		K concentration		Dry matter		K concentration		Dry matter		K concentration	
Year	2020	2021	2020	2021	2020	2021	2020	2021	2020	2021	2020	2021
Nitrogen (N)	101.49**	90.27**	3.01ns	17.91**	26.09*	39.1**	4.16ns	12.98*	100.03**	97.19**	0.53ns	3.37ns
Potassium (K)	9.19**	26.94**	6.75*	4.06ns	5.33*	22.53**	2.37ns	3.42ns	11.64**	43.82**	5.34*	5.01*
Cultivars (C)	16.79**	672.45**	97.31**	7.82**	11.85**	384.32**	7.5**	26.24**	29.21**	95.96**	35.31**	90.41**
N×K	5.49**	3.79*	1.05ns	3.02ns	3.05ns	11.39**	1.12ns	3.23ns	7.73*	15.89**	0.73ns	0.27ns
N×C	4.96**	35.81**	16.35**	1.60ns	7.58**	29.49**	0.82ns	1.68ns	14.48**	10.05**	1.77ns	0.81ns
K×C	1.28ns	5.14**	4.09*	0.21ns	0.37ns	1.91ns	0.70ns	3.15*	0.82ns	1.19ns	0.72ns	3.69*
N×K×C	1.46ns	1.87ns	1.58ns	8.26**	0.19ns	1.06ns	0.79ns	0.28ns	0.81ns	0.63ns	0.80ns	1.28ns

The ** and * indicate significant differences at the P<0.01 and P<0.05 levels, respectively, and ns represents no significant difference.

We found that an appropriate amount of N application will reduce K_c_ (**Figure 6**) and increased the KNI (**Figure 7**). Compared to low nitrogen treatment, high nitrogen treatment requires higher K to achieve maximum yield (**Figure 3**). **Figure 9** also showed that the KNI corresponding to the maximum relative yield increased with the increase of N fertilizer application. Therefore, the amount of K fertilizer application should be based on the amount of soil N content. Under low N condition, low K was required to achieve high yield; under medium N condition, medium K was required to achieve high yield. There was an optimal N/K ratio that maximizes the yield of sweetpotato. This interaction between N and K is important for the growth of sweetpotato (Ding et al., 2008). In the present study, N and K had a positive relationship for crop growth and accumulation, which is consistent with previous findings (Wang et al., 2016; Wang J et al., 2017). In 2020, K1 and K2 treatments had higher yield under the N0 mode, while K2 and K3 treatments have higher yield under the N0 mode. In 2021, K2 and K3 treatments had higher yield under the N0 mode, while K3 and K4 treatments have higher yield under the N1 mode. This is due to the concentrated rainfall from June to July in 2021, which increased the available N in the soil. So, the corresponding K demand to achieve high yield for two N mode in 2021 also further increased. Therefore, efficient production requires an understanding of the effects of N and K on sweetpotato, as well as accurate determination of K nutritional status based on K concentrations in distinct parts of the sweetpotato. This information can help to timely use N and K fertilizers, and reduce the pressure on the growth of sweetpotato.

## 5 Conclusion

Although the role of potassium fertilizer in sweet potato cultivation is well-known, there are still few studies on scientific diagnosis and application of critical potassium dilution curves in sweetpotato. We found that the required amount of K fertilizer to achieve maximum sweetpotato yield under high N conditions was greater than that under low nitrogen conditions. A potassium concentration dilution model for sweetpotato in different nitrogen fertilizer levels can be constructed to assess the nutritional status of sweetpotato. The critical K dilution curve (CKDC) of sweetpotato aboveground: K_c(N0)_=*5.30W^-0.463^*, R^2^ = 0.79, and K_c(N1)_=*4.23W^-0.298^*, R^2^ = 0.78, underground: K_c(N0)_=*1.38W^-0.125^*, R^2^ = 0.81, and K_c(N1)_=*1.32W^-0.132^*, R^2^ = 0.72;whole-plant: K_c(N0)_=*4.31W^-0.421^*, R^2^ = 0.80; Kc_(N1)_=*3.89W^-0.415^*, R^2^ = 0.79. The K_c_ and KNI can be used to diagnose the nutritional status of sweetpotato. The CKDC correctly identified the K status of the aboveground and underground parts and could be used as a reliable indicator of the potassium status of sweetpotato. Through the dilution curve to determine the nutritional status of K at the early growth stage, the management of N and K elements can be adjusted in time during the tuberous root enlargement stage, thereby increasing the yield. It provided a theoretical basis for the high-quality cultivation and K regulation of sweetpotato.

## Data availability statement

The original contributions presented in the study are included in the article/supplementary material. Further inquiries can be directed to the corresponding authors.

## Author contributions

WH: Designing the experiment, Participation in field trials, Writing; JL, YL, SC, LD, XX, YZ, MJ, YL: Participation in field trials; GL: Providing suggestions; ZL: Reviewing and Editing, Oversight and leadership responsibility for the research activity.
